# Employment Status and Health Literacy in Denmark: A Population-Based Study

**DOI:** 10.3389/ijph.2021.598083

**Published:** 2021-03-09

**Authors:** Ida W. Svendsen, Maria B. Damgaard, Carsten K. Bak, Henrik Bøggild, Christian Torp-Pedersen, Majbritt T. Svendsen, Gabriele Berg-Beckhoff

**Affiliations:** ^1^ CIMT - Centre for Innovative Medical Technology, Odense University Hospital, Odense, Denmark; ^2^ Unit for Health Promotion Research, Department of Public Health, University of Southern Denmark, Esbjerg, Denmark; ^3^ Department of Research and Development, University College South, Esbjerg, Denmark; ^4^ Public Health and Epidemiology Group, Department of Health Science and Technology, Aalborg University, Aalborg, Denmark; ^5^ Unit of Clinical Biostatistics, Aalborg University Hospital, Aalborg, Denmark; ^6^ Department of Cardiology, Centre for Clinical Research, North Denmark Regional Hospital, Hjørring, Denmark; ^7^ Hospital South West Jutland, Esbjerg, Denmark

**Keywords:** health literacy, occupation, economic public support, health promotion, cross sectional study, register data, HLS-EU-Q16, employment

## Abstract

**Objectives:** Examining whether specific population groups who are not working and those who have an employment have the same health literacy level.

**Methods:** Data were retrieved from a nationally representative cross-sectional study of the Danish population conducted with the health literacy questionnaire (HLS-EU-Q16) in 2016 and 2017. Socio-demographic characteristics were drawn from national registers. Odds ratio for the association between employment status and health literacy was estimated from logistic regression models, adjusted for socio-demographic characteristics. Probability weights were used to adjust for differences in responses.

**Results:** Logistic regression analyses showed that receiving unemployment benefits, social assistance, employment and support allowance, retirement pension and sickness benefit were significantly associated with having inadequate health literacy compared to being employed in any industry. The highest odds ratio for inadequate health literacy was present for receiving unemployment benefit OR = 1.78 (95% CI: 1.23–2.56).

**Conclusion:** Population groups not working and receiving economic public support have higher odds of inadequate health literacy competencies compared to those active in the labor force, considering age and socioeconomic factors. The result contributes to understanding health disparities in connection to occupational situation.

## Introduction

Over the last decades, the concept of health literacy has received increasing attention worldwide [1]. Health literacy is a complex phenomenon, concerning the ability to access, understand, appraise and apply health information, enabling the individual to promote and maintain health. The need for health literacy skills increases concurrently with the abundant health information available and the societal demand for the individuals to be active and involved regarding their health to prevent illness. It requires health competencies to be able to navigate in the complex health information and misinformation available and to make informed choices regarding health promotion and treatment [2]. According to WHO [3] limited health literacy is associated with less healthy choices, riskier health behavior, poorer health, suboptimal self-management, and more frequent hospitalisations, compared to individuals with a higher health literacy level. A study conducted in eight European countries found that almost half of the participants had limited health literacy [4]. Moreover, in Denmark, it is estimated that 10–20% of the population has difficulties in essential health literacy dimensions, such as the ability to understand health information well enough to know what to do, and the ability to actively engage with healthcare providers [5]. Results from a Danish study [6] found that nearly 40% of the study population faced difficulties in accessing, understanding, appraising, and applying health information as 8.18% presented with inadequate health literacy and 30.94% with problematic health literacy [6]. Inadequate health literacy is considered a public health challenge and is shown to have severe consequences, both for the individual but also at a societal level as it is directly and indirectly associated with poorer health outcomes [7, 8].

The individual’s health literacy competencies may be derived from educational or occupational experiences, emphasizing that people’s social environments throughout life have an influence on the development of health literacy competencies [9]. WHO [3] has emphasized the critical importance of the interface between organisations, the setting, and the individual for health literacy competences. Reviews on the link between health literacy and social context also highlights the different conceptual understanding and empirical operationalization of health literacy as a concept between disciplines, methods and research communities [10, 11]. Concepts such as social networks, social support, social ties, social inclusion and social practice are strongly represented in the theoretical literature but are used very loosely and widely in empirical research on health literacy [12]. This highlights the importance of considering the influence of the surroundings on the individual’s daily life and habits when developing health-promoting initiatives. The occupational environment constitutes a huge part of people’s life in industrialized societies as the average weekly working time for full-time employees in the EU is 37 h [13]. The individual’s occupational situation influences our physical and mental health. Unemployment is strongly associated with adverse health indicators such as higher mortality, unhealthy symptoms, and unfavourable lifestyles [14]. In Denmark, the universal welfare system contributes to a large degree for the differences in individual’s employment status by economic compensations securing equity and a flexible job market [15]. Danes who cannot work are supported through social insurances and economic public support provided by a public founded welfare state [15]. However, unemployed people miss out on repressive environments including interaction with colleagues and do not benefit from health promotion initiatives conducted at the workplace which can be factors that might increase the health literacy level. It may be noticed that within the employment groups the individuals different social and economic resources could also have an influence on their health.

As the future demographic changes entail substantial challenges in the health care system increasing the importance of the population to stay healthy, it is important to consider factors affecting the ability to maintain and promote a healthy life. Therefor it is also relevant to investigate whether working and the individual’s occupational situation influence the level of health literacy, impacting the ability to maintain and promote a healthy life [16]. The research-based knowledge on the association between employment status and health literacy is scarce but the evidence shows that different context-dependent status of employment are associated with health literacy [17–22]. Thus, this study aimed to identify whether the health literacy level differs dependent on the individuals’ employment situation by comparing different population groups who are not employed with those who are employed. The hypothesis was that people who receive economic public support from the state have a higher risk of having an inadequate health literacy compared to those who are employed.

## Method

### Study Design

Between December 2016 and February 2017, a random sample of the Danish population was invited to participate in a cross-sectional national representative survey. In total 15,682 adults in the age of 25 years or older were invited and 8,997 participated in the self-administrated electronic survey. All participants were drawn from the Danish Civil Registration System. Participants were recruited through an electronic e-mail system (e-Boks) used to administer information from public authorities and official institutions. To increase representativity, a random part of the study population (N = 1,082) were contacted by telephone. The sample was stratified for sex, age and geographical location. All material was provided in Danish. The response rate was 57.4% after removing observations with inadequate information or missing values a sample of 8,767 participants was considered for analysis.

### Measures

The survey included The European Health Literacy Questionnaire (HLS-EU-Q) which present items within four categories: 1) health literacy, 2) health behavior, 3) health risk indicators, and 4) perceived self-assessed health. The survey data was linked with information from national administrative registers at Statistic Denmark containing information on demographic characteristics, socioeconomic indicators, employment status and health status.

#### Health Literacy Variable

To measure health literacy the short 16-item version of the European Health Literacy Survey (HLS-EU-Q16) was applied. Each item was rated on a 4-point Likert scale [23]. For this study the item categories of the HLS-EU-Q16 were dichotomized into two categories “easy” (“fairly easy” or “very easy”) and “difficult” (“fairly difficult” or “very difficult”). Scale values were calculated as sum scores varying between 0 and 16, accounting for missing item responses. Participants who answered more than 14 of the 16 health literacy items were considered for analysis [24]. As it was relevant to consider the part of the population not having adequate level of health literacy the total health literacy score was dichotomized into a binary variable having the categories “adequate” (sufficient, score 13–16) and “inadequate” (limited and problematic, score 0–12) health literacy [23, 24].

#### Employment Status Variable

Information on employment status was obtained from the DREAM register [25]. This register allows description of people’s source of income and contains weekly information on type of employment and for those not working information on received public support are registered. The DREAM register is based on data from the Ministry of Employment and education and tax information [25]. Data from week 27 in year 2015 until week 26 in year 2016 was obtained. For each respondent, the numbers of weeks receiving a specific public economic support, or the main industrial sector was registered. Relevant publicly founded economic support included in this study were “unemployment benefits”, “social assistance”, “employment and support allowance”, “sickness benefit”, “retirement pension” and “voluntary early retirement pension” and “others” [26, 27]. See Table 1 for clarification of publicly founded economic support.

**TABLE 1:** Description of relevant public support for people not in work.

Public support classifications	Description
Unemployment benefits	A voluntary individual unemployment insurance which enables unemployment benefit for up to 2 years. Right to unemployment benefit requires, among other things, membership of an unemployment fund and that the person is registered as job seeking at a public jobcentre, fulfill a special employment requirement and are available to the job market. Furthermore, unemployment should not be self-inflicted
Social assistance	A public service which supports people who cannot otherwise support themselves and their families. All job opportunities should be utilized
Employment and support allowance	Employment and support allowance is an economic public support that ensures people with permanent and significantly reduced working capacity who are not able to support themselves, to have a supportive economic foundation
Sickness benefit	Sickness benefit is financial compensation for lost income from work which occurs as a result of sickness absenteeism. To be entitled to sickness benefit it is a condition that one is actively connected to the job market
Retirement pension	The retirement pension is a public pension, that ensures all Danish citizens who leave the job market at the statutory entitled time to have an income. The age for when people are entitled to retirement pension depends on birth year
Voluntary early retirement pension	With the voluntary early retirement scheme, an opportunity to withdraw completely or partially from the job market before you are entitled to retirement pension is possible if several conditions are fulfilled. Among the conditions is a continuous membership subscription over 30 years

#### Confounding Variables

Variables considered as confounders included age, sex, cohabitation, education, immigrant status and household income. Data were derived from either the survey or registers. Sex, age, and immigration status were obtained from the Danish Civil Personal Registration Registry which contains information on all citizens in Denmark who have a civil registration number [28]. Educational information was obtained from the Danish Education Registers [29]. The information was retrieved one year before survey completion. Average household income was obtained from the Danish Income Register [30] and was calculated based on income data for the years 2013–2015. Data was only used for analysis if observations had data for at least two years in the included period. When income was missing for one-year, average income was calculated based on the two years registered. Information on cohabitation was retrieved from the survey.

For analysis, the confounding variables were categorized as follows: Sex was treated as a binary variable; “female” and “male”. Age in years was coded in five categories: “25–34”, “35–44”, “45–54”, “55–64” and “65+”. The binary variable for immigration status includes; “Danish” and “foreigner” (including descendants of immigrants). Educational attainment defined as the highest achieved education level was coded in five categories: “Basic”, “high school/vocational”, “medium”, “high” and “unknown”. The medium-length education includes short and medium-length tertiary plus bachelor educations. Higher-length education consists of master-level and PhD-level educations. The average household income was coded as a binary variable with the categories; “below group average” and “above group average”. The average threshold was based on the sample data. The variable cohabitation was measured with six items coded as indicator variables; “living with partner or spouse”, “living with child or children below 16 years”, “living with parents”, “living alone”, “living with other adults >20 years” and “living with young (16–20 years)”. A new categorical variable was generated from the six indicator variables including the five categories “only partner”, “partner and child < 16”, “only child < 16”, “alone” and “others”.

### Ethics

Approval for data collection was granted by the Danish Data Protection Agency (j.no: 2008-58-0028) and collection was done in accordance with the Helsinki Declaration. Questionnaire-based and register-based studies do not require ethical approval according to Danish Legislation [31]. The survey includes information regarding information retrieval and voluntary completion. Providing information constituted an implied voluntary consent.

### Statistical Analyses

Chi-square tests were conducted to assess whether participant characteristics affects the probability of participation in the survey. Logistic regression was conducted to estimate the association between receiving public economic support and health literacy adjusting for the confounding effect of sex, age, immigration status, educational attainment, cohabitation, and household income. Weights were constructed by predicted probabilities from a logistic regression model using information from non-respondents and respondents according to sex, age, and education. Probability weights using survey set command were used in all analyses. Interactions of different strata of the variable sex were assessed by evaluating the change in model fit (pseudo R-squared) and *p*-value of the interaction term. The significance level was set at *p* < 0.05. Findings are presented as odds ratios (OR) with 95%-confidence intervals. All statistical analysis was conducted using the statistical software STATA/MP version 15.1.

## Results

Characteristics of the survey participants considered for analysis are displayed for responders receiving an online questionnaire (N = 7,862) and responders interviewed by telephone (N = 905) (Table 2). The effect of age groups, immigration status, education, and average yearly household income on responding were all significant (*p* < 0.001) for participants receiving an online questionnaire, which showed that specific characteristics were associated with responding the online survey. For the participants interviewed by telephone, there were no significant effects of sex, age groups and average household income per year on responding, but the effect of immigration status was significant (*p* = 0.004). Education data are not presented in Table 2 due to limited numbers (N < 5).

**TABLE 2:** Responder analysis stratified for online and telephone survey.

Characteristics	Online survey						Telephone survey				
	Participants^a^ N = 7,862		Non-participant^b^ N = 6,636			*p* ^c^	Participants N = 905		Non-participant N = 46		*p*
	N	%	N		%		N	%	N	%	
Sex											
Female	4,331	55.09	2,915		43.93	<0.001	467	51.60	27	58.70	0.348
Male	3,531	44.91	3,721		56.07		438	48.40	19	41.30	
Age groups											
25–34	950	12.08	1,668		25.14	<0.001	150	16.57	7	15.22	0.189
35–44	1,341	17.06	1,789		26.96		213	23.54	6	13.04	
45–54	1,951	24.82	1,493		22.50		189	20.88	11	23.91	
55–64	1,850	23.53	1,010		15.22		180	19.89	15	32.61	
65+	1,770	22.51	676		10.19		173	19.12	7	15.22	
Immigration status											
Danish	7,347	93.45	5,455		82.46		848	93.70	38	82.61	0.004
Foreigner	515	6.55	1,160		17.54	<0.001	57	6.30	8	17.39	
Average household income per year (DKK)											
Below group average	4,259	54.17		4.138	63.39		540	59.67	29	63.04	0.649
Above group average	3.603	45.83		2,390	36.61	<0.001	365	40.33	17	36.96	

Notes: Row percentages are presented

^a^ Cleaned data sample, participant characteristics (N = 230)

^b^ Missing values for non-participants (N = 3)

^c^ Chi^2^ analysis presented

The prevalence of inadequate health literacy was 39% (Table 3). The highest prevalence of inadequate health literacy was found for the group receiving unemployment benefit, around 54% and the lowest distribution was found for the group being on voluntary early retirement pension, around 32%.

**TABLE 3:** Prevalence of inadequate and adequate health literacy by relevant covariates (crude and weighted prevalence).

Characteristics	Adequate		Inadequate		Total	
	N (%)^a^	Weighted %^a^	N (%)^a^	Weighted %^a^	N (% of total)^b^	Weighted %^b^
Overall	5,461 (62.29)	61.02	3,306 (37.71)	38.98	8,767 (100)	100.00
Employment status						
Employed	3,589 (63.31)	62,16	2,080 (36.69)	37,84	5,669 (64.66)	67,27
Unemployment benefits	67 (49.26)	46,46	69 (50.74)	53,54	136 (1.55)	1,70
Social assistance	61 (45.19)	46,94	74 (54.81)	53,06	135 (1.54)	2,11
Employment and support allowance	163 (52.75)	51,19	146 (47.25)	48,81	309 (3.52)	3,48
Sickness benefit	129 (52.65)	51,64	116 (47.35)	48,36	245 (2.79)	2,97
Retirement pension	966 (64.36)	63,88	535 (35.64)	36,12	1,501 (17.12)	13,19
Voluntary early retirement pension	172 (68.25)	67,94	80 (31.75)	32,06	252 (2.87)	2,36
Others	314 (60.38)	59,08	206 (39.62)	0,92	520 (5.93)	6,92
Sex						
Female	3,197 (66.63)	65,40	1,601 (33.37)	34,60	4,798 (54.73)	50,22
Male	2,264 (57.04)	56,61	1,705 (42.96)	53,39	3,969 (45.27)	49,78
Age groups						
25–34	610 (55.45)	55,29	490 (44.55)	44,71	1,100 (12.55)	17,95
35–44	927 (59.65)	58,89	627 (40.35)	41,11	1,554 (17.73)	21,58
45–54	1,355 (63.32)	62,62	785 (36.68)	37,38	2,140 (24.41)	23,59
55–64	1,295 (63.79)	63,03	735 (36.21)	36,97	2,030 (23.16)	19,81
65+	1,274 (65.57)	65,23	669 (34.43)	34,77	1,943 (22.16)	17,07
Immigration status						
Danish	5,139 (62.71)	61,41	3,056 (37.29)	38,59	8,195 (93.48)	91,40
Foreigner	322 (56.29)	56,95	250 (43.71)	43,05	572 (6.52)	8,60
Education						
Basic school	842 (59.38)	58,17	576 (40.62)	41,83	1,418 (16.17)	17,90
High school/vocational	2,238 (60.26)	59,09	1,476 (39.74)	40,91	3,714 (42.36)	41,80
Medium	1,629 (67.01)	66,12	802 (32.99)	33,88	2,431 (27.73)	24,47
High	632 (62.76)	61,63	375 (37.24)	38,37	1,007 (11.49)	10,68
Unknown	120 (60.91)	61,23	77 (39.09)	38,77	197 (2.25)	5,15
Average household income per year (DKK)						
Below group average	2,901 (60.45)	59,05	1,898 (39.55)	40,95	4,799 (54.74)	56,25
Above group average	2,560 (64.52)	63,57	1,408 (35.48)	36,43	3,968 (45.26)	43,75
Cohabitation						
Spouse/partner	2,852 (63.93)	62,98	1,609 (36.07)	37,02	4,461 (50.88)	48,56
Partner and children	884 (60.10)	59,22	587 (39.90)	40,78	1,471 (16.78)	18,72
Only child	324 (55.67)	54,67	258 (44.33)	45,33	582 (6.64)	7,40
Alone	912 (60.24)	58,31	602 (39.76)	41,69	1,514 (17.27)	16,79
Others	489 (66.17)	64,69	250 (33.83)	35,31	739 (8.43)	8,54

^a^ Row percentages are presented

^b^ Column percentages are presented

Logistic regression (Table 4) showed that receiving unemployment benefits, social assistance, employment and support allowance, sickness benefit and retirement pension were significantly associated with having inadequate health literacy compared to being employed. For those receiving unemployment benefits, the association with having inadequate health literacy was significant with OR = 1.78 (95% CI:1.23–2.56) when adjusted for sociodemographic variables age group, sex, cohabitation, education group, immigration status and household and income. Furthermore, for the group receiving employment and support allowance, the association with having inadequate health literacy was also significant with an OR = 1.61 (95% CI:1.25–2.07).

**TABLE 4:** Odds ratio for employment status and inadequate health literacy (weighted data).

Employment status (Reference “employed”)	Crude OR (95% CI)	Adjusted for socio demographic variables^a^ OR (95% CI)
Unemployment benefits	1.89 (1.33–2.70)	1.78 (1.23–2.56)
Social assistance	1.85 (1.28–2.68)	1.49 (1.01–2.17)
Employment and support allowance	1.57 (1.24–1.98)	1.61 (1.25–2.07)
Sickness benefit	1.54 (1.18–2.00)	1.53 (1.17–2.01)
Retirement pension	0.93 (0.82–1.05)	1.34 (1.06–1.71)
Voluntary early retirement pension	0.77 (0.59–1.02)	0.97 (0.72–1.29)
Others	1.14 (0.93–1.39)	1.04 (0.85–1.27)

^a^ Adjusted for age group, sex, cohabitation, education group, immigrant status and household income.

## Discussion

This cross-sectional study found a difference in the prevalence of inadequate health literacy between the unemployed population receiving public support ranging from 32% to 54%, highest in the groups receiving unemployment benefit. The overall prevalence of inadequate health literacy was 39%. The analyses showed a significant association between all the categories of public support with having inadequate health literacy, except for voluntary early retirement pension. The results are consistent with our assumption and current body of evidence that the unemployed population has a higher likelihood of having inadequate health literacy [17–22, 32]. However, it should be noticed, that the setting and the measures differ from the current study.

Wu and colleagues [17] conducted a community based cross-sectional study in China in 2016 including 1,360 individuals aged 15–69 years to evaluate the prevalence of low health literacy. The study found that the group of unemployed were more likely to have low health literacy compared to those working as technical or professionals. A national cross-sectional study by Furuya and colleagues conducted in Japan [18] including 1,237 participants found that communicative/critical health literacy scores varied significantly depending on employment status and was lowest among the unemployed. In the study by Van de Heide and colleagues [20], the authors used a prediction model to investigate to what extent national health literacy levels could be validly estimated from socioeconomic and demographic characteristics. The study found that for those unemployed, the mean health literacy scores differed compared to those who were working, where the mean health literacy score was lower for those not working for both performance-based health literacy and self-assessed health literacy [20]. Furthermore, in the same study, it was found that working status was a significant predictor of performance-based health literacy where not working was significantly associated with having lower performance-based health literacy compared with working.

Health literacy is reliant on basic literacy skills but navigating in the complex health information also depends on the individual’s abilities to access, understand and apply the information, and furthermore, abilities to act, as this is an essential element for health promotion [33, 34]. Deduced from the literature the employment status seems to be associated with the level of health literacy. An explanation might be, that being employed provides good opportunities for general learning of literacy skills which positively affect the skills to derive meaning from texts and this might affect people’s performance on health literacy tasks [20]. However, the causal association between employment status and health literacy is still unclear.

Other factors influence the level of health literacy such as educational background [35]. Considering the connection between educational level and employment status, higher level of former employment was associated with higher health literacy levels [19]. Furthermore, a study by Wu and colleagues [17] found the prevalence of inadequate health literacy was particularly high for the group primarily engaged in manual labor. Hence it is relevant to consider the social gradient within the area of health literacy [36]. This implies that the type of work and work environment also have an impact on health literacy skills.

The results from this study have important implications. The results indicate that it might be important to look at the workplace as an arena for developing health literacy competencies. Therefor the association to the workplace is important when considering health literacy competencies as the employment status can be associated with health literacy independent of the individuals age, sex, cohabitation, education, immigrant status and household income. In the last century, a shift in health promotion strategies has occurred, of which a change is seen from the behaviorally focused health promotion approach to a healthy setting approach, such as the workplace, and how the setting can enhance healthy behavior [37]. The work environment should provide healthier possibilities for the employees encouraging them to engage in healthier activities and making healthier choices [38]. People outside the job market miss out on work environmental factors facilitating health literacy. Health policies must also be tailored to the vulnerable groups outside the job market, acknowledging that the citizens receiving economic public support are not a homogeneous group. When developing environmental health-promoting initiatives the strategies should consider the fact that almost 40% of the Danish population receiving economic public support have an inadequate health literacy level. Improving health literacy potentially can be a mean to reduce social inequality in health [3].

A reduction in social inequality in health can cause a greater part of the citizens to be included in the labor force assisting to reduce the costs to social and economic public support. Due to the expected demographic challenges, focus must be leveled at increasing the individual’s quality of life but also from a societal perspective promoting health and preventing mortality. This study stresses the importance of helping people into the job market as being unemployed have great costs for the individual, and though no causal association can be concluded this study indicates that employment might have a positive impact on health literacy and the health.

There are limitations of the study which should be acknowledged. For this population-based study, a cross-sectional study design was applied. Because of the simultaneous assessment of exposure and outcome, it is not possible to conclude a causal association [39]. Further research is needed to investigate the causal association between health literacy and receiving economic public support as it is unclear if employment status causes the health literacy level or if the level of health literacy affects employment status. For measuring health literacy, the HLS-EU-Q16 questionnaire was applied. As the questionnaire measures self-perceived health literacy there is a likelihood of respondents systematically over- or underestimating their experience which should be acknowledged as this could affect the results [40, 41]. Another limitation is related to data collection as the participation in questionnaires often appeals to a resourceful part of the population. To increase representativity, part of the data was retrieved by telephone interviews, but it is acknowledged as a limitation that the data for this part of the study is not robust. Strengths of the study are the large sample size and that the sample was stratified for sex, age and geographical location to ensure representability. Furthermore, telephone interviews were used to include the part of the population with limited literacy skills. To adjust for differences in responses probability weights were used in all analyses.

## Conclusion

There is an association between receiving economic public support and having inadequate health literacy, even when adjusting for important confounding variables. This contribute to understanding the health disparities in connection to occupational situation, health literacy and their relation to health. These results enable and facilitate preventive efforts for specific vulnerable groups in the society who are not active at the job market aiming at improving their health literacy skills. As the future demographic changes entail substantial challenges within the health care system and at the job market, the results facilitate health-promoting measures on a social level. Due to methodological limitations, no causal association can be concluded, and further research is needed to investigate the causal effect between health literacy and receiving economic public support.

## Data Availability

The data analyzed in this study is subject to the following licenses/restrictions: Register data not available for public use. Requests to access these datasets should be directed to mbdamgaard@health.sdu.dk.
